# [^68^ Ga]Ga-Prostate-Specific Membrane Antigen PET/CT: a novel method for imaging patients with hepatocellular carcinoma

**DOI:** 10.1007/s00259-020-05017-0

**Published:** 2020-09-03

**Authors:** Jolanta Kunikowska, Bartosz Cieślak, Beata Gierej, Waldemar Patkowski, Leszek Kraj, Marcin Kotulski, Krzysztof Zieniewicz, Leszek Królicki

**Affiliations:** 1 Nuclear Medicine Department, Medical University of Warsaw, ul. Banacha 1 a, 02-097 Warsaw, Poland; 2 Department of General Surgery, John Paul II Specialist Hospital, Nowy Targ, Poland; 3 Department of Pathology, Medical University of Warsaw, Warsaw, Poland; 4 Department of General, Transplant, and Liver Surgery, Medical University of Warsaw, Warsaw, Poland; 5 Department of Haematology, Oncology and Internal Medicine, Medical University of Warsaw, Warsaw, Poland; 6 Laboratory of Neurogenomics, Institute of Genetics and Animal Breeding, Polish Academy of Sciences, Jastrzebiec, Poland

**Keywords:** PSMA, HCC, [^68^Ga]Ga-PSMA-11, Hepatocellular carcinoma

## Abstract

**Purpose:**

Prostate-specific membrane antigen (PSMA) is not only highly expressed on the surface prostate cancer cells but is also elevated during angiogenesis in other cancer cell types, including hepatocellular carcinoma (HCC). This study aimed to evaluate the feasibility of using PET/CT imaging with [^68^Ga]Ga-PSMA-11 in HCC and its impact on patient management.

**Methods:**

Fifteen patients (13 men and two women; aged 55.6 ± 18.2 years) with HCC were enrolled in this prospective, single-institution study. All patients underwent contrast-enhanced MRI/CT, [^68^Ga]Ga-PSMA-11 PET/CT, and histopathological verification of lesions.

**Results:**

No radiopharmaceutical-related adverse events were noted. Visual interpretation showed increased accumulation of [^68^Ga]Ga-PSMA-11 in all HCC patients. The tumor-to-liver ratio (TLR) was 3.6 ± 2.1, and the maximal standardized uptake value (SUV_max_) was 13.5 ± 7.1. There were no significant differences in the SUVs or TLR between newly diagnosed and recurrent patients. No statistically significant relationship was found between serum concentration of tumor markers (i.e., AFP, CA 19-9, CEA) and PET parameters. Results of the [^68^Ga]Ga-PSMA-11 PET/CT changed the treatment strategy in five (33%) patients. PSMA staining showed visible heterogeneity in terms of intensity and distribution: the reaction was weak and only observed in a few vessels in pseudoglandular patterns of HCC, while it was homogeneously strong, with some hot spots, in trabecular patterns of HCC.

**Conclusion:**

[^68^Ga]Ga-PSMA-11 PET/CT can detect PSMA expression in vivo in patients with HCC and is useful for guiding treatment strategies. Further investigation of the clinical utility of this method in HCC is warranted.

## Introduction

Hepatocellular carcinoma (HCC) is the most common type of primary liver malignancy, and the fourth leading cause of cancer-related deaths worldwide [1]. The diagnosis of HCC is challenging and is based on invasive (tumor biopsy) or noninvasive methods (imaging techniques). Due to the difficulties associated with a biopsy (e.g., risk of bleeding, difficulty in targeting the tumor, or cancer cells seeding), imaging techniques have become the preferred way to confirm the diagnosis of HCC. Magnetic resonance imaging (MRI) and computed tomography (CT) are the most commonly used imaging modalities for the radiological diagnosis of HCC; however, their sensitivity is suboptimal, reaching approximately 70% for CT and 80% for MRI [2]. The assessment of HCC recurrence after primary treatment is even more difficult, because of post-therapy changes.

Another imaging option is positron emission tomography (PET) with fluorodeoxyglucose ([^18^F]FDG), which is widely used in various types of oncological diseases. However, as liver gluconeogenesis in well-differentiated HCC is comparable to normal, the sensitivity of [^18^F]FDG PET for detecting HCC is not better than that of conventional imaging (50–70%) [2]. Therefore, other PET tracers based on lipid metabolism have been proposed for the detection of HCC, including [^11^C]C-labeled acetate and [^18^F]F-choline. [^11^C]C-labeled acetate reportedly has improved sensitivity for low- and intermediate-grade HCC [3, 2]. Similarly, [^18^F]F-choline (which is a component of phosphatidylcholine, an essential element in cell membranes) has a similar sensitivity to [^11^C]C-labeled acetate, ranging from 75 to 87% [3, 2]. Moreover, prostate-specific membrane antigen (PSMA) has been implicated as a potential target for imaging studies in several tumor types.

PMSA, or glutamate carboxypeptidase II, was originally found to be specifically expressed in the epithelial cells of prostate cancer cells. However, recent studies have shown PSMA is also involved in angiogenesis in other cancer types, such as glioblastoma, gastric cancer, colon cancer, bladder cancer, HCC, clear cell renal carcinoma, breast cancer, ovarian cancer, melanoma, and mesothelioma [4–6]. As PSMA is only detected in histopathology during angiogenesis and not on normal vessels, it not only represents a potential novel target for diagnosis but also for therapy [7]. Milowsky et al. first demonstrated PSMA is a valid neovascular target in humans for treating advanced solid tumors by using an ^111^Indium-labeled monoclonal antibody (mAb) against PSMA (J591) [8]. Since then, a variety of PSMA-targeted radiotracers have become available, which have potential diagnostic and therapeutic applications [9, 10]. Among them, [^68^Ga]Ga-PSMA-HBED-CC (PSMA-11), [^18^F]DCFPyL, and [^18^F]PSMA-1007 are currently the most commonly used [9].

Since PSMA expression is associated with poor prognosis, the results of [^68^Ga]Ga-PSMA PET/CT have strong clinical implications [11]. However, to date, the use of [^68^Ga]Ga-PSMA PET/CT (mainly PSMA-11) for the diagnosis of HCC has only been reported in two studies [12, 13] and several case reports [14–18]. Therefore, this study aimed to evaluate the feasibility of PET/CT imaging with [^68^Ga]Ga-PSMA-11 in HCC and its impact on patient management.

## Patients and methods

### Patients

A total of 15 patients (13 men and two women, mean age 55.6 ± 18.2 years) were qualified for the study. All patients had a diagnosis of HCC confirmed histologically. Among them, ten patients were newly diagnosed; four patients had recurrence after transarterial chemoembolization (TACE); and one patient after hemihepatectomy and TACE. On the basis of Child-Pugh classification and Eastern Cooperative Oncology Group (ECOG) performance status score, 14 were Child-Pugh A/ECOG 0 or 1, and only one participant was Child-Pugh B and ECOG 2. The clinical characteristics of the patients are summarized in Table 1.

**Table 1:** The clinical characteristics of the patients

Number	Sex	Age	History	AFP (ng/ml *N* < 7)	CEA (ng/ml *N* < 3)	CA 19-9 (IU/ml *N* < 34)	HBV	HCV	Fatty liver	Liver cirrhosis	Child-Pugh	ECOG
1	M	72	Newly diagnosed	N	N	N	-	-	-	-	A	0
2	F	59	Newly diagnosed	N	13.6	66.4	-	+	+	+	A	0
3	M	73	Newly diagnosed	31,158	N	N	-	-	-	-	A	1
4	M	64	Newly diagnosed	78.4	10.6	N	-	+	-	+	A	1
5	M	69	After TACE	N	N	N	+	-	-	-	A	1
6	M	72	Newly diagnosed	N	N	N	-	-	+	-	A	1
7	M	61	After TACE	5433	15.2	N	+	+	-	-	A	0
8	M	28	After TACE	49.2	N	N	+	+	-	-	A	1
9	F	46	Newly diagnosed	N	N	51	-	-	-	-	A	0
10	M	66	Newly diagnosed	5738	N	N	+	-	-	-	A	1
11	M	53	Newly diagnosed	23.6	N	N	-	-	-	-	A	0
12	M	54	Newly diagnosed	N	N	N	+	-	-	+	A	1
13	M	18	After TACE	N	N	N	-	-	-	-	A	1
14	M	65	Newly diagnosed	50.2	5.2	40	-	-	-	+	B	2
15	M	71	After TACE	69,533	N	N	-	-	+	-	A	1

*AFP*, α-fetoprotein; *HCV*, hepatitis C virus; *HBV*, hepatitis B virus; *CEA*, carcinoembryonic antigen; *TACE*, transarterial chemoembolization; *ECOG*, Eastern Cooperative Oncology Group performance status

Included patients had one or more measurable target lesions (newly diagnosed or previously treated with TACE) based on Response Evaluation Criteria in Solid Tumors (RECIST). Within a 4-week interval, contrast-enhanced CT/MRI (ceCT/MRI) and [^68^Ga]Ga-PSMA-11 PET/CT were performed. Serum concentration of alpha-fetoprotein (AFP), carbohydrate antigen 19-9 (CA 19-9), and carcinoembryonic antigen (CEA) were assessed before [^68^Ga]Ga-PSMA-11 PET/CT.

Written informed consent was obtained from all patients before enrolment in the study. The study was approved by the Ethical Committee of Medical University of Warsaw (KB/2/A/2018).

### [^68^Ga]Ga-PSMA-11 PET/CT protocol and image interpretation

Radiopharmaceutical preparation of [^68^Ga]Ga-PSMA-11 and the imaging protocol was performed as previously described [5]. Briefly, PET/CT was performed from the skull to the thighs (3 min per bed position, three iterations, 21 subsets) on a Biograph 64 TruePoint (Siemens Medical Solutions Inc., USA) for 60 min post-injection of [^68^Ga]Ga-PSMA-11 (dose of 2 MBq/kg body weight). Image analyses were performed using the Siemens Workstation (Syngovia, MMWS, Siemens Medical Solutions Inc., USA).

On visual evaluation of lesions located in the liver, an uptake of [^68^Ga]Ga-PSMA-11 higher than that of the background liver was considered a positive result. In addition, “minor uptake” was defined as an uptake lower than that of the liver, “moderate uptake,” as a comparable or slightly higher than that of the liver, and “intense uptake” as significantly higher than that of the liver.

For quantitative analysis, the maximal standard uptake value (SUV_max_) and mean standard uptake value (SUV_mean_) of a positive lesion were measured on [^68^Ga]Ga-PSMA-11 PET/CT images with spherical volumes of interest (VOIs). Tumor-to-liver ratios (TLR) were calculated using the SUV_max_ of the lesion divided by the SUV_mean_ of the normal liver.

### Immunohistochemistry and PSMA evaluation

Tissue pieces obtained during surgery or core needle biopsy were cut into 4-μm sections from paraffin blocks and placed on glass slides. Next, the sections underwent deparaffinization with xylene and dehydration with gradient alcohol. Subsequently, antigen retrieval was performed at high temperature and pressure conditions in pH 9.0 citrate buffer. Sections were incubated with primary PSMA antibody (mouse mAb, Dako/Agilent, Clone 3E6; dilution 1:50). A prostate cancer sample was used as a positive control. The EnVision FLEX was used for the visualization of staining. All sections were then counterstained with hematoxylin. All procedures were performed according to the manufacturer’s instructions.

Vascular PSMA expression was semiquantitatively scored based on staining intensity (weak vs. strong) and distribution. Lesions with no detectable PSMA expression were scored as “0” (negative); lesions with PSMA staining in 1–50% of vasculatures were scored as “1” (low PSMA expression); and lesions with PSMA staining in > 50% of vasculatures were scored as “2” (high PSMA expression). Endothelial expression of PSMA in the tumor vasculature was confirmed by CD31 staining, a well-established endothelial cell marker.

The evaluation of immunohistochemical staining was carried out by two experienced pathologists, who were blinded to clinical data.

### Statistical methods

All variables were evaluated for their distribution pattern. Means and standard deviations were used to summarize the patients’ characteristics. Calculations were done on Excel for MAC (version 16.28, 2019 Microsoft). The McNemar test of correlated properties was used to compare the results of [^68^Ga]Ga-PSMA-11 and ceCT/MRI imaging. In all cases, a *p* value < 0.05 was considered statistically significant.

All statistical analysis was performed using GraphPad PRISM 5 (GraphPad Software Inc.) software.

## Results

### Toxicity reporting

No adverse events related to the use of the diagnostic radiopharmaceutical were recorded.

### Visual and semiquantitative [^68^Ga]Ga-PSMA-11 PET/CT image analysis

Forty-four lesions in 15 patients demonstrated an uptake in [^68^Ga]Ga-PSMA-11 PET/CT on visual evaluation. Among them, 38 lesions were located in the liver, five in the bones, and one in the peritoneum. Based on visual analysis, intense uptake of [^68^Ga]Ga-PSMA-11 was shown in 30 lesions, with moderate uptake in 11 lesions. Only three bone lesions in the ribs showed a minor uptake of [^68^Ga]Ga-PSMA-11 with an SUV_max_ of 2.2–2.4, which was lower than that observed in the liver.

The lesions were also assessed quantitatively using SUV_max_, SUV_mean_, and TLR (as summarized in Table 2). The SUV_max_ was 13.5 ± 7.1 (range: 2.2–29.3) and the SUV_mean_ was 6.5 ± 2.6 (range: 2.1–13.7). The TLRs obtained from [^68^Ga]Ga-PSMA-11 PET/CT varied between 1.6 and 10.9, with a mean TLR of 3.6 ± 2.1. For lesions located in liver, the SUV_max_ was 14.7 ± 6.7 (range: 5.5–29.3) and the SUV_mean_ was 6.9 ± 2.5 (range: 3.3–13.7). For metastatic lesions, the SUV_max_ was 6.1 ± 4.0 (range: 2.2–12.0) and the SUV_mean_ was 3.1 ± 1.1 (range: 2.3–4.6).

**Table 2:** [^68^Ga]Ga-PSMA-11 uptake of tumors and surrounding liver tissue

Patient no.	SUV_max_ of the tumor/metastasis	SUV_mean_ of the tumor/metastasis	SUV_max_ of the liver tissue	SUV_mean_ of the liver tissue	TLR (SUV_max_ of the tumor/SUV_mean_ of the liver)
1	16.3	5.7	6.7	4.5	3.6
2	8.0	4.9	5.0	3.4	2.4
3	29.3	6.7	3.2	2.7	10.9
4	7.0	3.3	5.0	3.1	2.3
5	17.6	7.5	5.6	4.4	4.0
	8.4	5.5	5.6	4.4	1.9
6	13.6	5.9	6.2	4.5	3.0
	7.3	5.6	6.2	4.5	1.6
	8.1	4.6	6.2	4.5	1.8
7	25.4	5.5	4.8	3.3	7.7
	6.3	4.5	4.2	3.3	1.9
	5.5	3.8	4.2	3.3	1.7
8	7.8	4.5	5.0	3.3	2.4
	6.8	4.7	5.0	3.3	2.1
9	15.5	7.9	7.1	4.5	3.4
10	17.8	8.5	6.3	3.4	5.2
	8.1	5.8	6.3	3.4	2.4
	7.4	4.5	6.3	3.4	2.2
	9.3	4.5	6.3	3.4	2.7
	12.0	8.0	6.3	3.4	3.5
11	24.3	6.0	5.9	3.2	7.6
12	11.4	4.4	5.0	3.1	3.7
13	22.7	8.7	6.4	4.3	5.3
	9.6	5.4	6.4	4.3	2.2
	11.6	6.2	6.4	4.3	2.7
	19.6	10.3	6.4	4.3	4.6
	22.5	8.0	6.4	4.3	5.2
	18.2	9.6	6.4	4.3	4.2
	20.1	12.2	6.4	4.3	4.7
	17.3	6.0	6.4	4.3	4.0
	11.3	6.6	6.4	4.3	2.6
	20.6	12.4	6.4	4.3	4.8
	23.6	13.7	6.4	4.3	5.5
	16.9	8.3	6.4	4.3	3.9
	23.6	9.6	6.4	4.3	5.5
	12.1	8.4	6.4	4.3	2.8
	8.9	5.9	6.4	4.3	2.1
14	12.7	7.2	6.1	3.9	3.3
15	24.5	7.8	3.8	2.9	8.4

*SUV*_*max*_, maximal standardized uptake value; *SUV*_*mean*_, mean standardized uptake value; *TLR*, tumor-to-liver ratio

### Newly diagnosed vs. previously diagnosed patients

Among the newly diagnosed patients, there were 19 lesions with an SUV_max_ of 11.2 ± 7.2 (range: 5.5–25.4) and an SUV_mean_ of 5.3 ± 1.8 (range: 3.3–8.5); patients after TACE had 25 lesions, with an SUV_max_ of 15.2 ± 6.6 (range: 3.4–29.3) and an SUV_mean_ of 7.4 ± 2.7 (range: 3.8–13.7). The mean TLR obtained on [^68^Ga]Ga-PSMA-11 PET/CT for newly diagnosed vs. after TACE was 3.2 ± 2.5 vs. 3.8 ± 1.9, respectively. There were no significant differences in the SUVs or TLRs between newly and previously diagnosed patients.

### Correlation between ceCT/MRI and [^68^Ga]Ga-PSMA-11 PET/CT images

[^68^Ga]Ga-PSMA-11 PET images were fused with the corresponding abdominal ceCT/MRI images using the semi-automatic image registration with manual adjustment for correct alignment. Visual interpretation of PET showed an increased accumulation of [^68^Ga]Ga-PSMA-11 in the contrast-enhancing parts of the tumor visible on ceCT/MRI. Simultaneously, there was no observed uptake in the necrotic parts of the tumor.

**Fig. 1:**
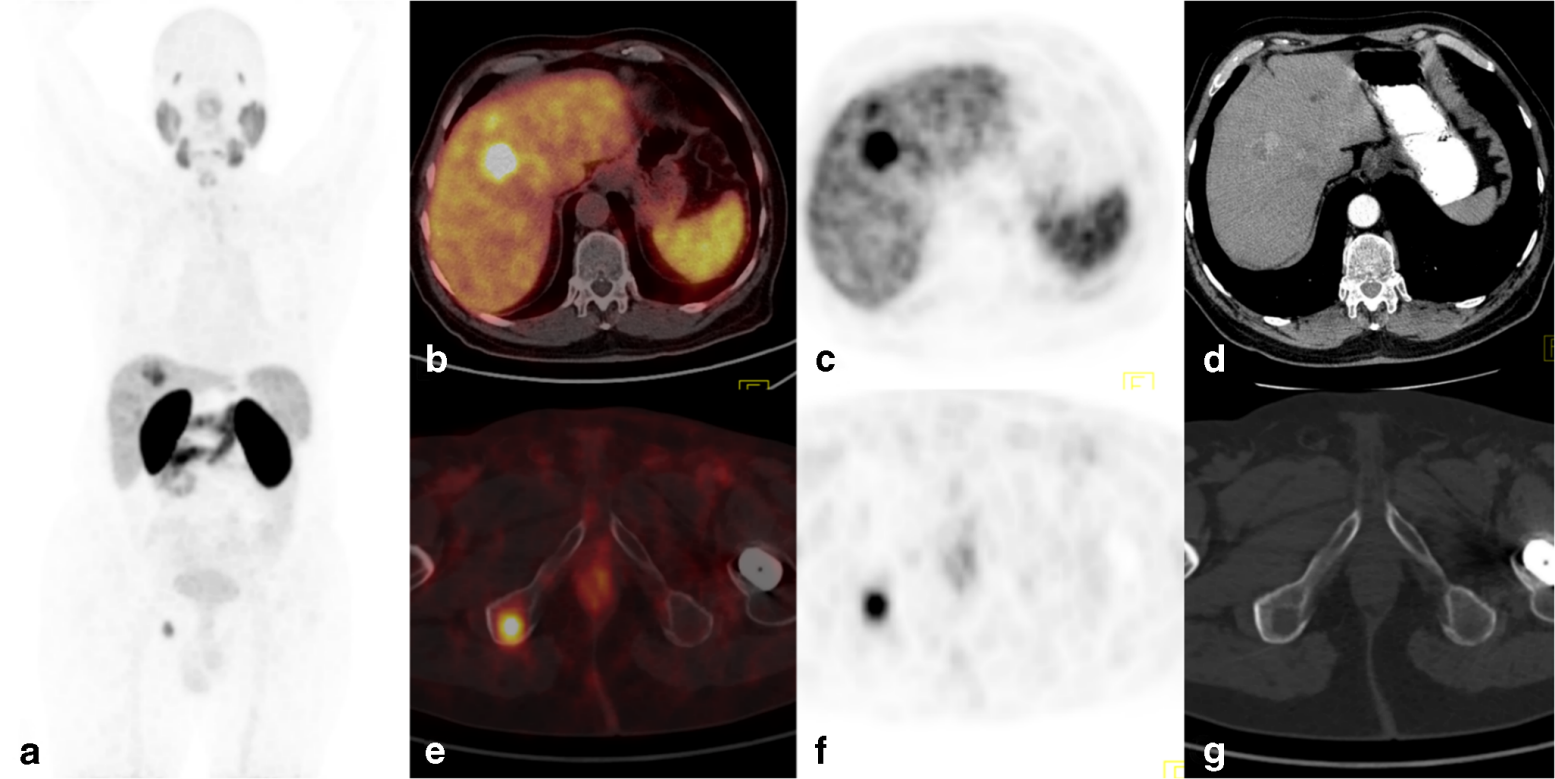
[^68^Ga]Ga-PSMA-11 PET/CT images of a 66-year-old man with hepatitis B virus (HBV) infection (patient no. 10). Follow-up revealed elevated CEA and AFP levels. On CT, there were two lesions suspicious of HCC. The [^68^Ga]Ga-PSMA-11 PET/CT images showed multiple liver tumors (SUV_max_ 7.4–17.8) and distant metastases in the bones (right VI rib, SUV_max_ of 2.2; right ischium, SUV_max_ of 12.0). **a** Maximum intensity projection image (MIP). **b**, **e** CT. **c**, **f** PET. **d**, **g** Fused axial PET/CT. Core needle biopsy of the liver revealed hepatocellular carcinoma grade 2. The patient was disqualified from surgery

[^68^Ga]Ga-PSMA-11 PET/CT showed more lesions in the liver than ceCT/MRI (38 vs. 29; *p* = 0.007). The median size of the liver lesions on ceCT/MRI was 23 mm (range: 8–167 mm). Meanwhile, the median size of lesions not visible on ceCT/MRI but visualized on PET was 8 mm (range: 2–10 mm). Notably, two out of the 15 patients had unexpected extrahepatic metastatic bone lesions on [^68^Ga]Ga-PSMA-11 (Fig. 1).

Following abdominal imaging ceCT/MRI, all patients had initially qualified for surgical resection or TACE. Indeed, the results of [^68^Ga]Ga-PSMA-11 PET/CT altered the previously planned treatment in five patients. In particular, due to additional foci revealed in the liver, two patients were disqualified from planned surgical resection, and one patient was disqualified from TACE. The remaining two patients were disqualified from surgery as [^68^Ga]Ga-PSMA-11 revealed disseminated disease: one due to bone metastases (Fig. 1), and the second due to an 8-mm peritoneum metastasis (with an SUV_max_ of 11.2), which was also found on ceCT but without contrast enhancement and deemed not related to cancer. All these patients were discussed at a multidisciplinary board meeting and finally qualified to chemotherapy.

### Compliance with tumor markers

Serum AFP positivity rate was 53% (8/15 patients) in the study group. The SUV_max_ was 15.0 ± 5.4 (SUV_mean_ 7.5 ± 2.6) in patients with a serum AFP level within the normal range, and 11.5 ± 8.5 (SUV_mean_ 5.2 ± 1.4) in those with an elevated AFP level. Meanwhile, serum CA 19-9 results were positive in 20% of patients (3/15). The SUV_max_ was 14.4 ± 7.0 (SUV_mean_ 6.8 ± 2.6) in patients with a normal CA19-9 level, and 12.1 ± 3.8 (SUV_mean_ 6.4 ± 2.1) in those with elevated CA 19-9 levels. Furthermore, 27% (4/15) of patients had a positive serum CEA measurement result. The SUV_max_ was 14.5 ± 6.4 (SUV_mean_ 7.1 ± 2.5) in patients with a normal CEA, and 10.4 ± 7.6 (SUV_mean_ 4.4 ± 0.9) in those with an elevated CEA level. No statistically significant relationships were found between the serum concentrations of AFP, CA 19-9, CEA, and PET parameters.

### Histopathology and immunohistochemistry

All patients had histopathological confirmation of the diagnosis. Metastatic lesions in the bone and peritoneum were confirmed by clinical follow-up and imaging examinations. The use of [^68^Ga]Ga-PSMA-11 PET/CT did not show false-positive findings based on histopathology or follow-up.

Sample specimens for PSMA staining were available in six patients. The immunochemistry showed intense intra-tumoural microvessel staining for PSMA and no staining in epithelial tumor cells. There was visible intra-tumoural heterogeneity of the reaction in terms of intensity and distribution. A strong correlation between the histopathological pattern and PSMA expression was found. In trabecular patterns of HCC, the reaction was homogenously strong in vessels with a tendency to create hot spots. However, in pseudoglandular patterns of HCC, the reaction was weak; only a few vessels showed a slightly stronger reaction than in the sinusoids of the surrounding non-tumourous liver parenchyma. In a well-differentiated hepatocellular neoplasm with uncertain malignant potential (HUMP), no staining was identified in vessels, yet there was a positive reaction in the sinusoids that was stronger than in the non-tumourous liver tissue. Additionally, in one patient with hepatitis B, there was a strong reaction for PSMA in intracellular hyaline bodies, and for PSMA, the reaction was weakly positive in the sinusoids in the surrounding non-tumourous liver parenchyma, but negative in the blood vessels and epithelial tumor cells.

**Fig. 2:**
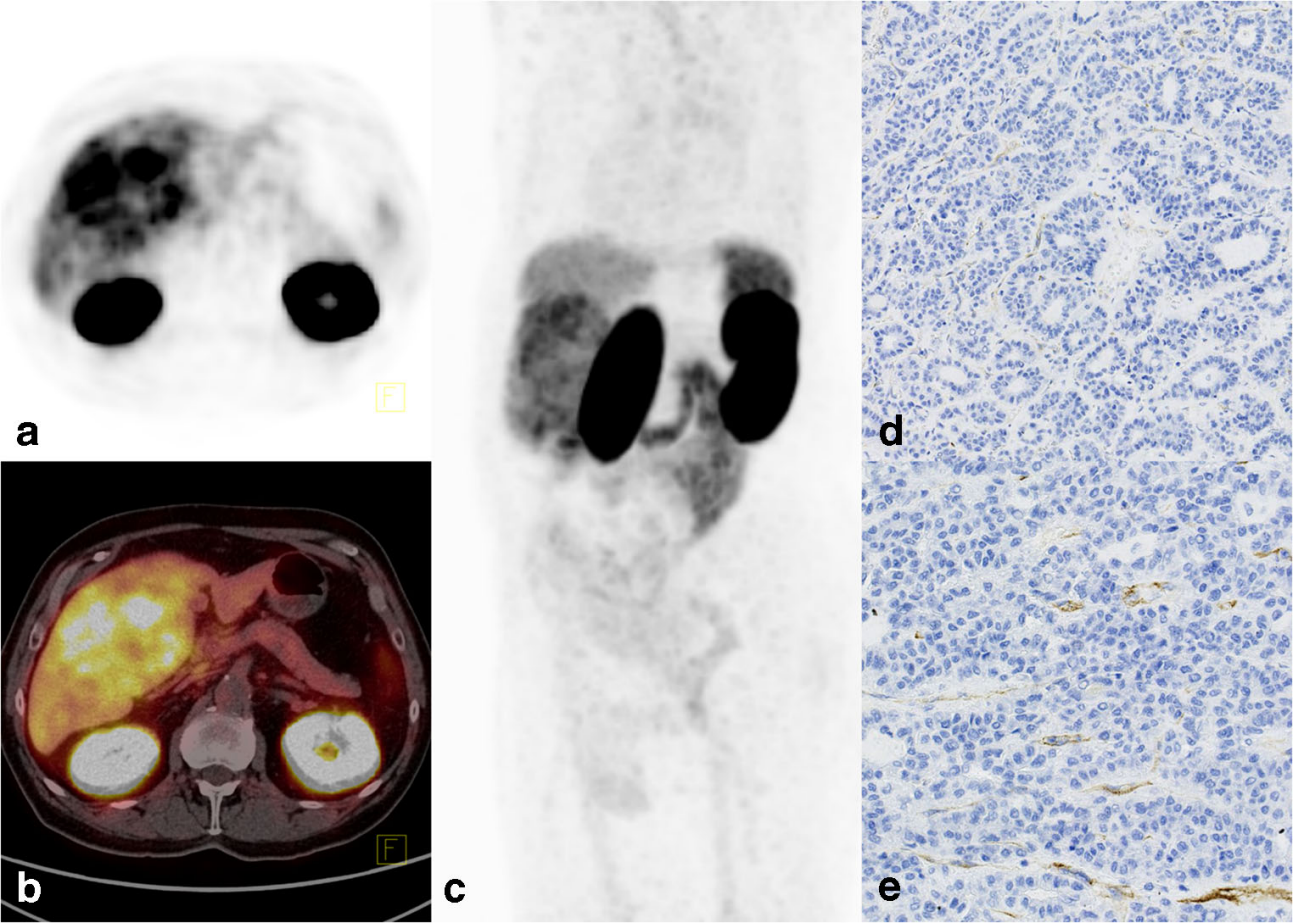
[^68^Ga]Ga-PSMA-11 PET/CT images of a 72-year-old man without a history of liver disease (patient no. 1). An ultrasound was performed due to abdominal pain, which revealed a huge (~ 100 mm) lesion in the liver suspicious of hemangioma. The MRI revealed a contrast-enhanced tumor with a suspicion of HCC or hemangioma. The laboratory tests for AFP, CEA, and CA19-9 were normal. The [^68^Ga]Ga-PSMA-11 PET/CT images showed high accumulation in the tumor with an SUV_max_ of 16.3 and TLR of 3.6. **a** PET. **b** Fused axial PET/CT. **c** Maximum intensity projection image (MIP). Histopathology revealed hepatocellular carcinoma grade 2, with a pseudoglandular pattern. Immunohistochemistry showed **d** weak PSMA expression in the tumor-associated vasculature of HCC (× 10) and **e** slightly stronger PMSA expression in some vessels than in the sinusoids of the surrounding healthy liver parenchyma (× 20)

**Fig. 3:**
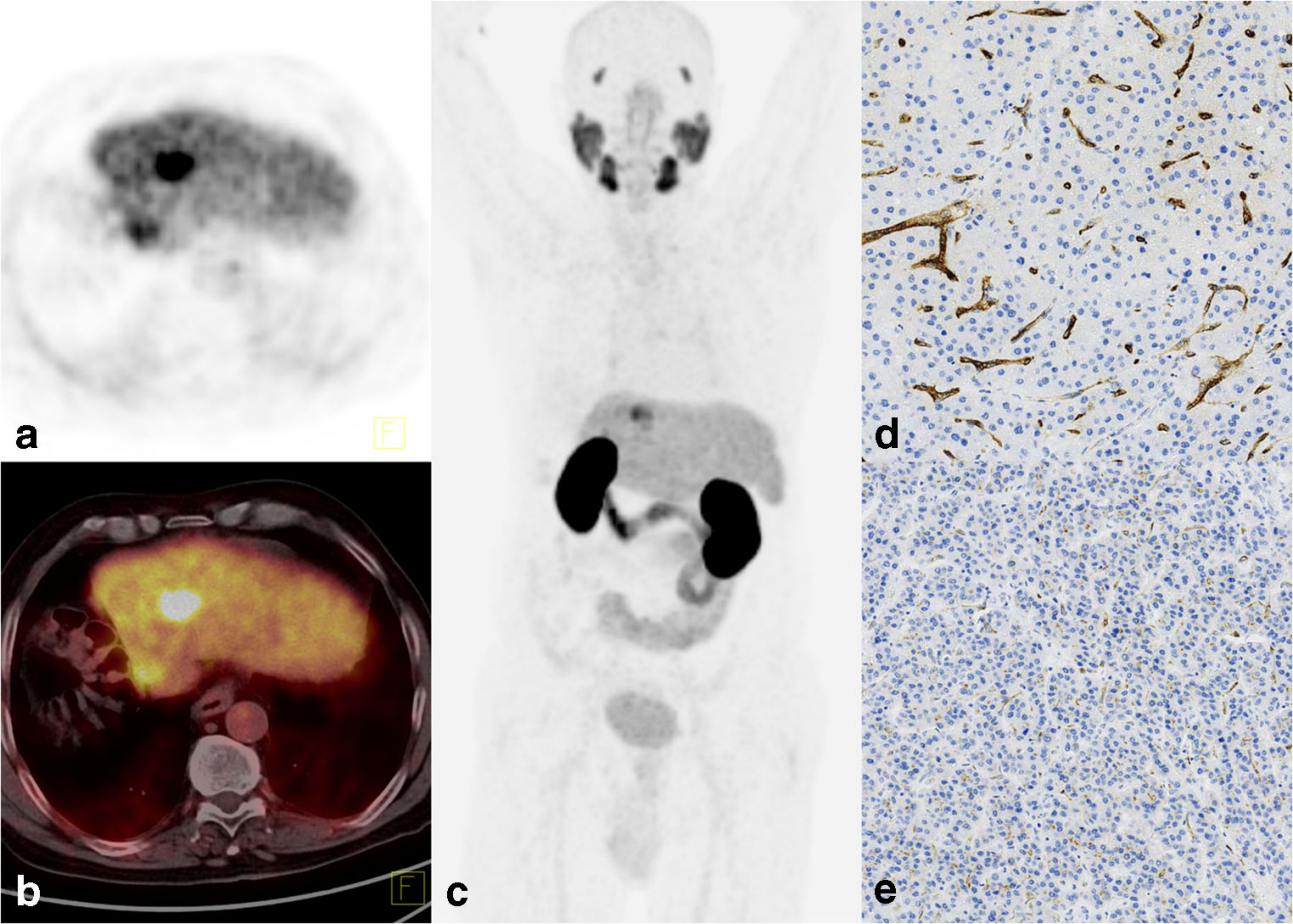
[^68^Ga]Ga-PSMA-11 PET/CT images of a 69-year-old man with hepatitis B virus (HBV) infection after hemihepatectomy due to HCC (patient no. 5). Follow-up MRI revealed a 20-mm lesion suspicious of HCC with normal cancer marker levels. [^68^Ga]Ga-PSMA-11 PET/CT images showed focal uptake with an SUV_max_ of 17.6 and TLR of 4.0, as well as a tiny lesion in the cutting line with an SUV_max_ of 8.4 and a TLR of 1.9. **a** PET. **b** Fused axial PET/CT. **c** Maximum intensity projection image (MIP). Histopathology revealed hepatocellular carcinoma grade 1, with a mixed pseudoglandular and trabecular pattern. Immunohistochemistry showed **d** strong neovascular staining for PSMA in a trabecular pattern of HCC (× 10) and **e**) a weak reaction in the pseudoglandular part, with only a few vessels showing slightly stronger staining (× 10)

**Fig. 4:**
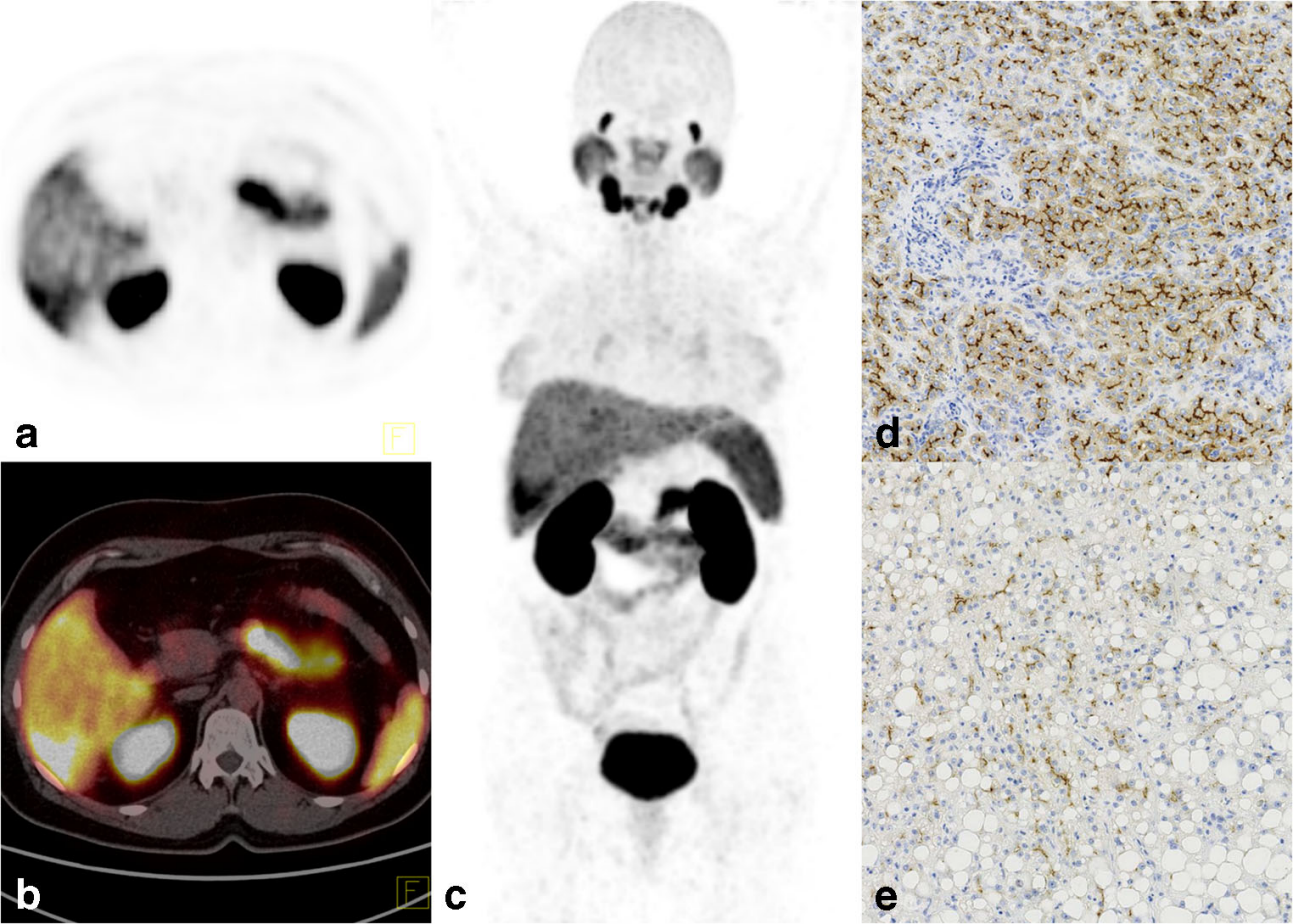
[^68^Ga]Ga-PSMA-11 PET/CT images of a 46-year-old woman without a history of liver disease (patient no. 9). An ultrasound was performed due to abdominal pain, which revealed a lesion in the liver suspicious of hemangioma. The MRI revealed a contrast-enhanced tumor with a suspicion of HCC or atypical hemangioma. Laboratory tests showed elevated CEA and CA 19-9 levels. The [^68^Ga]Ga-PSMA-11 PET/CT images showed high accumulation in the tumor with a SUV_max_ of 15.5 and a TLR of 3.4. **a** PET. **b** Fused axial PET/CT. **c** Maximum intensity projection image (MIP). Histopathology revealed a well-differentiated hepatocellular neoplasm with uncertain malignant potential. Immunohistochemistry showed **d** intense staining for PSMA within the sinusoids in the compact part with inflammatory infiltrates of the tumor (× 10) and **e** weaker staining in tumor areas with steatosis (× 10)

Regardless of PSMA expression visible in the immunochemistry, the [^68^Ga]Ga-PSMA-11 PET/CT showed an increased uptake in all patients (Table 3). The examples of tracer uptake patterns and immunochemistry staining are shown in Figs. 2, 3, and 4.

**Table 3:** [^68^Ga]Ga-PSMA-11 uptake and PSMA staining pattern on immunohistochemistry

Number	Tumor (SUV_max_)	Tumor (SUV_mean_)	TLR	Pattern	PSMA staining
1	16.3	5.7	3.6	Pseudoglandular	Weak reaction in few vessels
2	7.0	3.3	2.3	Trabecular	Strong reaction in vessels
3	17.6	7.5	4.0	Mix pseudoglandular and trabecular	Strong reaction in vessels in the trabecular pattern and a weak reaction in a few vessels in the pseudoglandular part
4	15.5	7.9	3.4	HUMP: well-differentiated hepatocellular neoplasm with uncertain malignant potential	No vessels staining, reaction in sinusoids (stronger than in the surrounding liver parenchyma)
5	17.8	8.5	5.2	Trabecular	Strong reaction in vessels
6	11.4	4.4	3.7	Mix pseudoglandular and trabecular	Strong reaction in vessels in the trabecular pattern and a weak reaction in a few vessels in the pseudoglandular part

*SUV*_*max*_, maximal standardized uptake value; *SUV*_*mean*_, mean standardized uptake value; *TLR*, tumor-to-liver ratio

## Discussion

Because [^18^F]F-choline PET/CT examination is not available in our hospital and [^18^F]FDG examination is not reimbursed for HCC, another imaging method for HCC is required. [^68^Ga]Ga-PSMA-11 was recently introduced for the PET/CT imaging of patients with prostate cancer, but incidental uptake has been described in other cancers, including reports on HCC [12–18].

The present pilot study showed encouraging preliminary results confirming the clinical utility of [^68^Ga]Ga-PSMA-11 PET/CT in patients with HCC. All histopathologically proven cases of HCC showed an increased tracer uptake. The results of [^68^Ga]Ga-PSMA-11 PET/CT were compared with ceCT/MRI for a total of 44 lesions detected in 15 patients. However, [^68^Ga]Ga-PSMA-11 PET/CT can be used for functional whole-body imaging, which may change their management. One of the most important findings is the clinical impact of the chosen imaging method on patient management. From a clinical point of view, the HCC treatment strategy includes surgery; local chemoembolization; and, in cases of disseminated disease, chemotherapy. [^68^Ga]Ga-PSMA-11 PET/CT results assisted the multidisciplinary team in choosing the optimal therapy. Functional and whole-body imaging PET revealed significantly more lesions than ceCT/MRI, enforcing a crucial change in the treatment strategy in 33% of patients. These findings indicated the superiority of [^68^Ga]Ga-PSMA-11 PET/CT over conventional imaging modalities.

The use of [^68^Ga]Ga-PSMA-11 PET/CT in HCC has been evaluated in two prior studies [12, 13]. Kesler et al. [12] studied seven mostly newly diagnosed HCC patients, and Kuyumcu et al. [13] included 19 patients with advanced disease. Both studies compared [^68^Ga]Ga-PSMA-11 with [^18^F]FDG. Kesler et al. [12] in newly diagnosed patients described more lesions revealed by [^68^Ga]Ga-PSMA-11 PET/CT than in [^18^F]FDG. Kuyumcu et al. [13] in patients with previously established diagnosis of HCC, so with a more advanced disease, showed that the utility of both tracers was comparable. In contrast, our cohort was more comprehensive, including patients with both types of disease status (i.e., those with a new diagnosis and a recurrence) and all had histopathological confirmation of disease. Importantly, patients with disease recurrence in our study had longer history of disease after TACE.

A previously published limited immunohistochemistry study showed that 74–80% of HCC tumors presented moderate-to-strong staining for PSMA, while completely negative staining was seen in only 4–26% of samples [11, 19]. All studies showed PSMA expression in the neovascular structures [11, 12, 19]. Tolkach et al. [19] also reported PSMA expression on the canalicular membrane within the liver, but no expression was detected in the vasculature of the surrounding normal liver tissue. Similarly, in our cohort, we confirmed that PSMA is specifically expressed in the tumor-associated vasculature.

Although one patient with hepatitis B showed also strong reaction for PSMA in the intracellular hyaline bodies, this was likely a nonspecific reaction. Additionally, a well-differentiated hepatocellular neoplasm with uncertain malignant potential showed no staining for PSMA in the vessels, but we observed bright, diffuse expression in the sinusoids. We also found a positive reaction in the vessels in both pseudoglandular and trabecular patterns of HCC, albeit of different intensity. The reaction was generally weak in pseudoglandular patterns of HCC, with slightly stronger spots demonstrated only in a few vessels. In contrast, it was homogenously strong with a tendency to create hot spots in trabecular patterns of HCC. These differences have not yet been described in the literature. However, further studies with increased sample numbers are required to confirm observed dependency.

All HCC patients in our cohort had an increased uptake of [^68^Ga]Ga-PSMA-11, which is likely related to the abovementioned elevated PSMA expression in the intra-tumoural microvessels. Indeed, the observed tracer uptake in tumors was 3.6-fold higher than that in the surrounding liver, with the mean TLR for advanced and for newly diagnosed patients equaling 3.8 and 3.2, respectively. Other authors described a similar increase in tracer uptake in tumors compared with the surrounding liver; i.e., it was 3.3-fold higher in patients with advanced HCC and 3.8-fold higher in those who were newly diagnosed [12, 13].

Our semiquantitative analysis revealed a mean SUV_max_ of 13.5, which is similar to that described by Kesler et al. for the enhanced part of the tumor in CT (i.e., an SUV_max_ of 13.2; range: 9.5–15.4) [12]. However, the SUV_max_ for HCC was higher in another cohort of patients with advanced disease (i.e., 17.4 ± 9; range 3.8–36.9) [13]. Nonetheless, we found no statistically significant difference in the uptake of [^68^Ga]Ga-PSMA-11 between newly diagnosed patients and those with recurrence after TACE.

Our findings, and those of others, suggest PSMA expression may be useful for therapeutic and diagnostic applications outside of prostate cancer. Indeed, Jiao et al. [11] reported that patients with HCC who had high vascular PSMA expression tend to have shorter overall survival. Therefore, [^68^Ga]Ga-PSMA-11 PET/CT could potentially be used as a prognostic factor in HCC [11]. The selective expression of PSMA in the tumor-associated vasculature in cases of HCC, and not in the tumor cells themselves, indicates PSMA may be an effective antiangiogenic treatment target for HCC [20–22]. In particular, the use of PSMA-targeted radioisotopes may be an effective HCC therapy. However, it has been suggested that the uptake of radiolabeled compounds by the microvascular endothelium (and not by tumor cells) may not be optimal (i.e., characterized by a quick washout). Despite this, recent pioneering dosimetry data of [^177^Lu]Lu-PSMA-617 treatment in other tumors with angiogenesis-associated PSMA expression (e.g., glioblastoma multiforme) has proven the possibility of such targeted treatment, without quick washout of radiolabeled compounds [23]. Moreover, in HCC, we found most extrahepatic metastatic sites (except bone) showed a higher uptake of tracer than the liver (with a TLR of 3.6 ± 2.1), indicating [^177^Lu]Lu-PSMA-617 may be a useful treatment. Indeed, according to the European Association of Nuclear Medicine (EANM) guideline based on the reported outrider phase II trial on [^177^Lu]Lu-PSMA-617, the required SUV_max_ at dominant sites of tumor involvement should be at least 1.5-fold higher than the baseline SUV_mean_ [^68^Ga]Ga-PSMA-11 of the liver for qualification for therapy [20, 21]. Therefore, the criterion of TLR > 1.5 used for the qualification of [^177^Lu]Lu-PSMA therapy would have been fulfilled by all patients in our cohort.

The current pilot study has major limitations to be disclosed. First, the number of patients is limited. The relatively small number of cases included is the result of strict inclusion criteria: only patients with histopathologically proven disease were qualified. The second limitation is the limited available sample tissue for immunohistochemistry staining.

Overall, we observed an increased uptake of [^68^Ga]Ga-PSMA-11 in all patients with histopathologically proven HCC, and found a different model of PSMA staining in pseudoglandular pattern and trabecular patterns of HCC. However, these promising results need further evaluation to confirm the observed dependency. The important future task is to calculate sensitivity, specificity, and other validating measures of [^68^Ga]Ga-PSMA-11 PET/CT in HCC. In particular, the performance characteristics of [^68^Ga]Ga-PSMA-11 PET/CT in HCC should be assessed.

## Conclusion

The use of [^68^Ga]Ga-PSMA-11 PET/CT could detect PSMA expression in vivo in patients with histopathologically proven HCC. Therefore, [^68^Ga]Ga-PSMA-11 PET/CT represents a potential novel imaging modality for patients with HCC. Immunochemistry confirmed the expression of PSMA in vitro in all patients with an increased uptake of [^68^Ga]Ga-PSMA-11 on PET/CT. Moreover, the use of [^68^Ga]Ga-PSMA-11 PET/CT resulted in a crucial change in the treatment in 33% of patients. Therefore, the potential role of [^68^Ga]Ga-PSMA-11 PET/CT in patients with HCC should be evaluated in further studies.

## Data Availability

Raw data are available from the corresponding author on reasonable request.
